# Radial Thermoelectric Model for Stranded Transmission Line Conductors

**DOI:** 10.3390/s23229205

**Published:** 2023-11-15

**Authors:** Jordi-Roger Riba

**Affiliations:** Campus Terrassa, Universitat Politècnica de Catalunya, Rambla Sant Nebridi 22, 08222 Terrassa, Barcelona, Spain; jordi.riba-ruiz@upc.edu; Tel.: +34-937398365

**Keywords:** stranded conductor, thermal model, transient conditions, radial temperature gradient, simulation, dynamic line rating

## Abstract

Bare-stranded conductors play a critical role in the efficiency and safe operation of transmission lines. The heat generated in the interior of the conductor is conducted radially to the outer surface, creating a radial thermal gradient. The radial temperature gradient between the core and the surface depends on multiple factors, such as stranding, number of layers, current level, electrical resistance and the effective radial thermal conductivity. Therefore, the radial temperature model must be considered when developing accurate conductor models. Such models are particularly important in the development of dynamic line rating (DLR) approaches to allow the full current carrying capacity of the conductor to be utilized while ensuring safe operation. This paper develops a radial one-dimensional thermoelectric model for bare-stranded conductors used in transmission lines. The accuracy of the proposed model is determined by experimental tests performed on three conductors.

## 1. Introduction

Global demand for electricity has been growing steadily, and projections indicate that this trend will continue. According to projections by the International Energy Agency (IEA), the share of electricity in final energy consumption is expected to increase from 20% today to more than 50% by 2050 [1]. The efficiency, reliability and availability of power lines will become increasingly important as electrification continues [2], so operators need reliable methods to operate overhead transmission lines at maximum capacity [3] without compromising their safety. Conductor sag is a key parameter for the safe operation of overhead transmission lines [4], as it determines the ground clearance. By limiting the maximum operating conductor temperature, sag, ground clearance [2,5] and conductor integrity [6] can be maintained at safe levels. Transmission lines occasionally operate at maximum design ratings [7]. Due to various factors, the construction of new transmission lines is very problematic today, so other options to increase the capacity of existing power lines are being analyzed, such as replacing existing aluminum conductors steel reinforced (ACSRs) with high-temperature low-sag (HTLS) conductors or by applying dynamic line rating (DLR) strategies. Unlike static line rating (SLR), which applies conservative ratings based on the worst-case weather conditions (emissivity = 0.8, high annual ambient temperature, wind speed of 0.5 m/s and solar irradiance of 1000 W/m^2^ [7]), DLR is based on real-time weather ratings [8,9,10]. By considering actual values of the solar irradiance, wind speed and direction and ambient temperature, combined with the application of DLR line models, a more realistic line rating can be determined [11,12], allowing the full capacity of the line to be utilized [13].

DLR approaches require the measurement of several variables, including conductor current and temperature and weather-related variables, such as ambient temperature, solar irradiance and wind speed and direction [14,15]. Next, the conductor heat balance equation is solved using methods such as those presented in Cigré [11], IEEE [12] or IEC [16], which also require knowledge of all conductor dimensions and material properties of the conductors. By solving the dynamic transient heat equation, it is possible to determine the maximum conductor rating. The DLR must ensure the stability of the transmission line at high operating currents while respecting the maximum allowable temperature of the conductor to ensure that safe limits of sag and annealing effect on the aluminum material are not exceeded [17]. For this purpose, it is essential to have a detailed thermoelectric model of the conductor, capable of predicting not only the surface temperature but also the higher internal temperatures of the conductor.

However, most of the models published in the technical literature assume a homogeneous temperature distribution within the conductor [11,12,16], which is a simplification of reality. Although this assumption may be correct in many cases, it can lead to non-negligible deviations in others, e.g., when considering ACSR conductors with at least three aluminum layers and high current densities [18], so that differences between 10 °C and 25 °C [12] up to 30 °C [19] have been reported. Various authors have obtained formulas for determining the radial temperature distribution of power conductors [11,12,17,19,20]. However, they make different assumptions, such as steady-state operation or uniform radial electrical conductivity, and do not allow the development of a transient model by themselves. In addition, most of these references provide experimental results, but they lack the development of a transient model capable of determining the thermal behavior of the conductor, taking into account the radial temperature distribution.

The temperature of the conductor results from a thermal balance between the heating terms (Joule and solar heating) and the cooling terms (radiative and convective cooling). If the heating terms are greater than the cooling terms, the conductor heats up; otherwise, it cools down. In these cases, the difference between the heat input and the heat output is the stored power term (see Section 2 for more details). While the radiative and convective cooling terms depend on the surface temperature, the Joule heating and stored power terms depend on the internal or average temperature of the conductor. Therefore, existing models that assume a homogeneous temperature distribution lack this detailed analysis because they assume no difference between the surface temperature and the center temperature of the conductor.

Therefore, a transient thermoelectric radial model of bare-stranded conductors is required. This can be achieved by modifying the standard methods used for calculating the maximum current carrying capacity of a conductor described in the standards [11,12,16] to account for the higher core temperature of the conductor [17].

The novelties and contributions of this paper are as follows. First, it focuses on improving the thermoelectric transmission line conductor models described in the international standards [11,12,16] to consider the radial temperature distribution in order to have a more accurate picture of the temperature distribution within the conductor. Second, this paper proposes an accurate and fast one-dimensional thermoelectric conductor model that considers the radial temperature distribution and assumes that the heat flows radially from the center of the core to the outer surface. Third, results showing how the radial temperature distribution affects the conductor heating are also presented. Fourth, the paper also presents experimental results of three types of conductors, namely, an all-aluminum alloy conductor (AAAC), an aluminum conductor steel reinforced (ACSR), and an aluminum conductor composite core (ACCC), a type of high-temperature low-sag (HTLS) conductor. Experimental results allow the accuracy and usefulness of the radial model presented in this paper to be evaluated.

The remainder of the paper is organized as follows. Section 2 develops the transient heat transfer equations and their components. Section 3 details the proposed one-dimensional discretization of the problem to determine the radial temperature distribution within the conductor. Section 4 details the importance of effective radial thermal conductivity in the proposed model. Section 5 develops the equations to solve the proposed radial model. Section 6 describes the transmission line conductors analyzed and the experimental setup. Section 7 validates the proposed radial model from the experimental results. Finally, Section 8 concludes this paper.

## 2. Transient Heat Balance Equation

**Figure 1:**
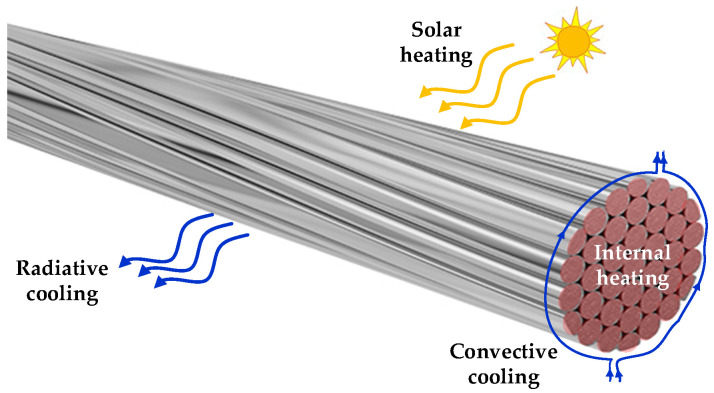
Heating and cooling terms in a stranded conductor according to Equation (1).

This section develops the different terms in the transient heat balance equation that describe the thermoelectric behavior of a bare conductor [11,12,21],

(1) $$(P_{internal}+P_{solar})-(P_{conv}+P_{rad})=mc_{p}(T)\frac{dT}{dt}=P_{stored}\;[W/m]$$

where *m* (kg/m) is the conductor mass per unit length, *c_p_*(*T*) (J/(kg °C)) is the specific heat capacity of the conductor material, *T* (°C) is the average temperature of the conductor, *P_internal_* (W/m) is the term related to the internal power losses generated in the conductor per unit length, *P_solar_* (W/m) is the solar heat gain per unit length, *P_conv_* (W/m) is the convective cooling term per unit length and *P_rad_* (W/m) is the radiative cooling per unit length. For a better understanding, the terms in (1) are shown in Figure 1.

According to (1), under thermal equilibrium, the total heat loss is equal to the total heat gain. Under transient conditions, where there is no thermal equilibrium, the difference between the total heat gain and the total heat loss is the neat heat stored in the conductor [11]. The various terms in (1) are developed in Section 2, but *P_internal_ = i*^2^(*t*)*r_AC_*(*T*) (W/m) is the internal heating, where *i*(*t*) (A) is the RMS value of the current measured at time instant *t* (s) and *r_AC_* (W/m) is the alternating current (AC) resistance per unit length of the conductor.

While the terms *P_internal_ = i*^2^(*t*)*r_AC_*(*T*) and *P_stored_* = *mc_p_*(*T*)d*T*/d*t* depend on the average conductor temperature *T*, the terms *P_conv_* and *P_rad_* depend on the conductor surface temperature. Note that *P_internal_ = i*^2^(*t*)*r_AC_*(*T*) is usually the main source of heating, and the AC resistance depends on the temperature distribution inside the conductor. In addition, effects such as creep, annealing and sag of the conductor are strongly affected by the radial temperature distribution within the conductor [22]. Therefore, accurate thermoelectric conductor models must take into account the temperature distribution within the conductor.

### 2.1. Stored Heat

Under transient conditions, i.e., without thermal equilibrium, the heat stored in the conductor per unit length can be expressed as,

(2) $$P_{stored}=mc_{p}(T)\frac{dT}{dt}\;[W/m]$$

### 2.2. Internal Heating

ACSR conductors consist of an inner core of galvanized steel strands and several layers of aluminum strands wound helically around the steel strands [23]. While the core provides the mechanical strength [24], the aluminum strands carry most of the current [25]. Due to the specific configuration and the magnetic properties of the steel core, the ac magnetic flux causes eddy currents, hysteresis losses in the core and a redistribution of the currents in the aluminum layers [18,26], increasing the conductor’s AC resistance [24]. The internal losses *P_internal_* can be determined as [8,21,27,28],

*P_internal_* = *P_Joule_* + *P_mag_* + *P_redis_* = *i*^2^(*t*)*r_AC_* (W/m) (3)

The internal losses have various components, namely the Joule losses (*P_Joule_*), the core losses due to the hysteresis and eddy current effects (*P_mag_*) and the redistribution losses (*P_redi_*_s_). According to (3), the internal losses are related to the AC resistance per unit length of the conductor *r_AC_* (Ω/m) and the electric current *i*(*t*).

In the case of conductors without a magnetic core, this results in *P_internal_* = *P_Joule_* since *P_mag_ = P_redis_* = 0.

The electrical resistivity *ρ_e_* of the conductor changes with temperature according to,

*ρ_e_*(*T*) = *ρ_e_*_,*To*_ [1 + *α_AC_*(*T* − *T*_0_)] (4)

where *T*_0_ is the reference temperature (usually 293.15 K), *T* (K) is the average temperature of the conductor temperature, and *α*_AC_ (K^−1^) is the temperature coefficient of the resistivity under alternating current. The temperature dependence of the conductor resistance per unit length *r_AC_*(*T*) follows a similar law,

*r_AC_*(*T*) = *r_AC_*_,*To*_ [1 + *α_AC_*(*T* − *T*_0_)] (5)

Since *P_internal_* is by far the largest source of heat in power conductors [8,27], *r_AC_* is an important parameter for building accurate thermoelectric models of conductors because it accounts for magnetic and eddy current effects. Note that *r*_AC_ can be calculated from *r*_DC_ (it is typically found in the manufacturers’ datasheets) using the procedure described in [11], or it can be measured directly as described in [28]. In this paper, *r*_AC_ is measured directly.

### 2.3. Natural Convective Heat Loss

According to IEEE Std. 738 [12], assuming a cylindrical conductor of outer diameter *D_c_* (m), the natural convective heat loss per unit length can be calculated as follows,

*P_conv_* = *h*π*D_c_*(*T_s_* − *T_air_*) (W/m) (6)

where *T_s_* (K) and *T_air_* (K) are the conductor surface temperature and air temperature, respectively, and *h* (W/(m^2^K)) is the heat transfer coefficient, which can be calculated under natural convection conditions (no wind, worst case) according to IEEE Std. 738 [12] as,

(7) $$h=\frac{3.645}{\pi }\rho_{air}^{0.5}D_{c}^{-0.25}{\left(T_{s}-T_{air}\right)}^{0.25}$$

Note that the air density *ρ_air_* (kg/m^3^), which depends on the conductor height *H* (m), and the temperatures of the air and the outer surface of the conductor, *T_air_* (K) and *T_s_* (K), respectively, can be determined as [12],

*ρ_air_* = (1.293 − 1.525 × 10^−4^*H* + 6.379 ×·10^−9^*H*^2^)/[1 + 1.835 ×·10^−3^(*T_air_* + *T_s_*)] (kg/m^3^) (8)

### 2.4. Radiative Heat Loss

The radiative heat loss per unit length of the conductor can be calculated as [11],

*P_rad_* = *πεD_c_σ_B_*[*T_s_*^4^ − *T_air_*^4^] (W/m) (9)

where *ε* (-) is the dimensionless emissivity coefficient and *σ_B_* = 5.67037 × 10^−8^ W/(m^2^K^4^) is the Stefan–Boltzmann constant. The effect of this term becomes very important at high conductor temperatures.

### 2.5. Solar Heat Gain

The solar heat gain *P_solar_* (W/m) can be calculated as [11,16],

(10) $$P_{solar}=\alpha Q_{sr}D_{c}\;(W/m)$$

where *α* ∈ [0, 1] and is the absorptivity of the conductor and *Q_sr_* (W/m^2^) is the total solar irradiance. This formulation is convenient because global radiation meters are now affordable, reliable and widely available. According to (10), the effect of solar heating increases with the conductor diameter [29]. Note that according to Kirchhoff’s law of thermal radiation, *α* ≈ *ε*. This equality also holds for non-equilibrium processes where radiative emission is produced exclusively by microscopic thermal transitions [30].

## 3. One-Dimensional Discretization of the Radial Temperature Distribution Problem

**Figure 2:**
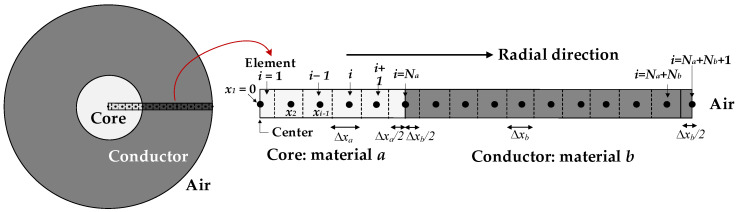
One-dimensional radial discretization of the conductor with *N*_a_ and *N*_b_ radial elements of width Δ*x*_a_ and Δ*x*_b_ (m). A transition node is placed at the core/conductor boundary and at the conductor/air boundary. The temperature of the central point of each node is assumed to be the mean temperature of the real element it represents.

Although the conductors are made of aluminum strands with very good conductivity, they have a radial temperature distribution that cannot be neglected, especially when the conductors are operated at high temperatures. The terms *P_internal_* and *dT*/*dt* in the heat balance Equation (1) depend on the average internal temperature of the conductor, while the terms *P_conv_* and *P_rad_* depend on the surface temperature. Since these two temperatures are different, it is necessary to know the radial temperature distribution of the conductor. To determine the radial temperature distribution, it is assumed that the heat flows radially from the center of the core to the outer surface. Due to the cylindrical geometry, a symmetric radial distribution can be assumed, allowing the problem to be discretized in one dimension, as shown in Figure 2.

The one-dimensional radial discretization shown in Figure 2 consists of *j* = 1, …, *n* = *N_a_* + *N_b_* + 1 nodes. It is assumed that the nodal temperatures of a generic node *i* can be determined from the nodal temperatures of the two adjacent nodes to the left and right sides. While the first node *j* = 1 is located in the center of the conductor (center of the core), the last node *j* = *n* is located in the periphery of the conductor (outer surface). The discretized form of the heat transfer problem expressed by (1) is solved through the radial axis as,

(11) $$a_{i}^{j}T_{{}_{,}i-1}^{j+1}+b_{i}^{j}T_{{}_{,}i}^{j+1}+c_{i}^{j}T_{{}_{,}i+1}^{j+1}-d_{i}^{j}=0$$

where *a_i_^j^*, *b_i_^j^*, *c_i_^j^* and *d_i_^j^* are constant coefficients, *i* is the index related to the radial steps and *j* is the index related to the time steps. Thus, *T_i_^j^* = *T*(*i*Δ*x*,*j*Δ*t*) represents the average temperature of node *i* calculated at the *j* time step, where Δ*x* is the radial step and Δ*t* is the time step.

Note that (11) determines the node temperatures from the temperatures of the adjacent right and left nodes. Equation (11) can be expressed in terms of a tri-diagonal matrix containing the node temperatures as,

(12) $$\begin{array}{l} \left[\begin{array}{ccccc} b_{1}^{j} & c_{1}^{j} & & & \\ a_{2}^{j} & b_{2}^{j} & c_{2}^{j} & . & \\ & a_{3}^{j} & b_{3}^{j} & c_{3}^{j} & .\\ c_{n}^{j}=0 & & & & a_{n}^{j} \end{array}\begin{array}{c} a_{1}^{j}=0\\ c_{n-1}^{j}\\ b_{n}^{j} \end{array}\right]\left[\begin{array}{c} T_{1}^{j+1}\\ T_{2}^{j+1}\\ T_{3}^{j+1}\\ .\\ T_{n}^{j+1} \end{array}\right]=\left[\begin{array}{c} d_{1}^{j}\\ d_{2}^{j}\\ d_{3}^{j}\\ .\\ d_{n}^{j} \end{array}\right] \end{array}$$

Using the tri-diagonal matrix algorithm (TDMA) approach [31,32], the node temperatures of all radial nodes are obtained for each simulation time *j*Δ*t*. Next, the Thomas algorithm, a simplified version of Gaussian elimination that speeds up the computational process [33], can be used to solve (12). Further details on the discretization procedure and the application of the TDMA algorithm can be found in [31,32].

### 3.1. Discretized Heat Balance Equation in a Generic Discrete Radial Element

According to Fourier’s law, the heat flow rate per unit length in the radial direction in an isotropic cylindrical conductor of diameter *D_c_* (m) can be written as follows,

(13) $$P_{conduction}=-k\pi D_{c}\frac{dT}{dx}\;(W/m)$$

where *k* (W/(mK)) is the thermal conductivity of the material, and d*T*/d*x* (K/m) is the derivative of the absolute temperature in the radial direction. Equation (13) can be expressed in discrete form as,

(14) $$P_{conduction}=-k\pi D_{c}\frac{\Delta T}{\Delta x}\;(W/m)$$

**Figure 3:**
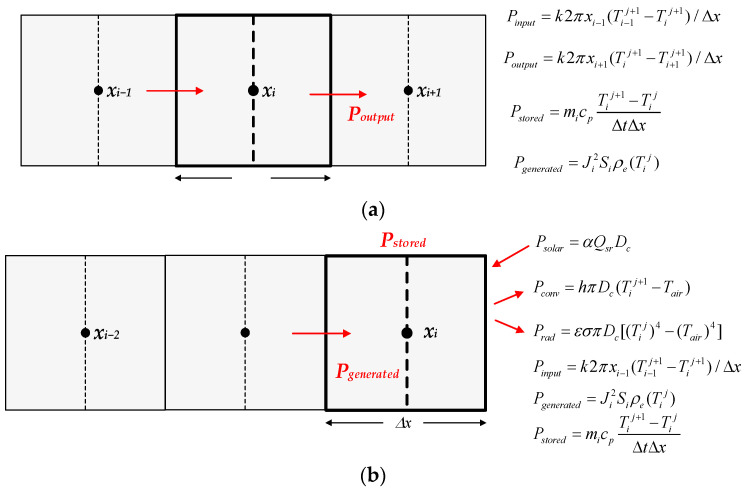
Heat balance. (**a**) In a generic interior element. (**b**) In the outermost element.

Figure 3 shows the heat balance in a generic inner element and in the outermost element,

### 3.2. TDMA Formulation

To solve the problem, it is necessary to determine the electrical resistance and the electrical current through each discrete element. Therefore, it is necessary to determine the electrical resistivity *ρ_e_*_,*i*_ (Ω·m) and the current density *J_i_* = *I*/*S_i_* (A/m^2^) in each discrete element, where *I* (A) and *S_i_* = π*x_i_*^2^ (m^2^) are the electrical current flowing through the conductor and the cross-sectional area of the discrete element number *i*, and *x_i_* (m) is the radial position of node *i*. The resistance per unit length of this discrete element can be calculated as *r_i_* = *ρ_e_*_,*i*_/*S_i_* (Ω/m).

The heat transfer equation in a generic element (see Figure 3) can be expressed as,

*P_stored_* = *P_generated_* + *P_input_* − *P_output_* (15)

Since *P_generatred_* = (*J_i_S_i_*)^2^*r_i_* = *J_i_*^2^*S_i_ρ_e_*(*T_i_^j^*), the discretized heat transfer equation in a given internal element (there is no solar heating or convective and radiative cooling) can be expressed as,

(16) $$m_{i}c_{p}\frac{T_{i}^{j+1}-T_{i}^{j}}{\Delta t\Delta x}=J_{i}^{2}S_{i}\rho_{e}(T_{i}^{j})+\frac{k2\pi x_{i-1}(T_{i-1}^{j+1}-T_{i}^{j+1})}{\Delta x}-\frac{k2\pi x_{i+1}(T_{i}^{j+1}-T_{i+1}^{j+1})}{\Delta x}\;(W/m)$$

where *c_p_* (J/(kgK)) is the specific heat capacity of the material, *m_i_* = *ρS_i_*Δ*x* (kg) is the mass of the *i*-th discrete element and *T_i_^j^* (K) is the absolute temperature of that discrete element at time *t* = *j*Δ*t* (s), while *ρ* (kg/m^3^) is the volumetric mass density of the material.

### 3.3. Particularities of ACSR Conductors

**Figure 4:**
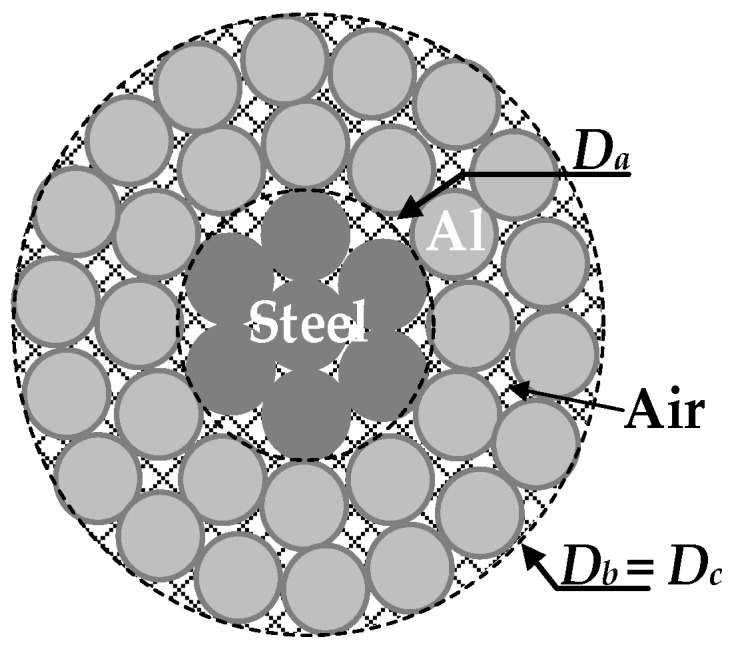
Cross-section of an ACSR conductor, showing the steel core and the conductor parts.

ACSR conductors have a core composed of stainless steel strands and a conductor body composed of aluminum strands, as shown in Figure 4.

Due to the higher electrical conductivity of aluminum, most of the current (approximately 98% [25]) flows through the aluminum strands. The following procedure is used to determine the current densities in the steel core strands and in the aluminum strands. First, the geometric cross-section of the core and the conductor parts can be calculated as *S_a_* = π·*D_a_*^2^/4 and *S_b_* = π·*D_b_*^2^/4 − *S_a_* (m^2^), respectively, where *D_a_* (m) and *D_b_* = *D_c_* (m) are the outer diameters of the core and the aluminum part, respectively. It should be noted that the geometric cross-sections *S_a_* and *S_b_* do not coincide with the effective cross-sections of the steel and aluminum parts, *S_eff_*_,*a*_ and *S_eff_*_,*b*_, respectively, due to the air voids and air gaps existing between the strands.

The mass per unit length of the core and the conductor parts, *m_a_* (kg/m) and *m_b_* (kg/m), respectively, are typically specified by the conductor manufacturer. Thus, the mass density of the core and aluminum parts can be determined as *ρ_a_* = *m_a_*/*S_a_* (kg/m^3^) and *ρ_b_* = *m_b_*/*S_b_* (kg/m^3^), respectively.

Since the current density through the steel strands is very low, their electrical resistivity can be taken as the tabulated value, *ρ_e_*_,*a*_ = 6.9 ×·10^−7^ Ω·m. However, since the current density through the aluminum strands is high and *S_b_* > *S_eff_*_,*b*_, the electrical resistivity of the conductor part used in the calculations can be determined as follows,

(17) $$r_{AC}=\frac{r_{a}r_{b}}{r_{a}+r_{b}}=\frac{\frac{\rho_{e,a}}{S_{a}}\cdot \frac{\rho_{e,b}}{S_{b}}}{\frac{\rho_{e,a}}{S_{a}}+\frac{\rho_{e,b}}{S_{b}}}\;[\Omega /m]\;\to \rho_{e,b}=r_{AC}\frac{\rho_{e,a}S_{b}}{\rho_{e,a}-r_{AC}S_{a}}$$

where *r*_AC_ (Ω/m) is the AC resistance per unit length of the conductor, which includes the effects of eddy currents and core losses. Note that (17) is derived from the parallel between the electrical resistances per unit length of the core *r_a_* and the conductive part *r_b_*. Once the resistivity *ρ_e_*_,*a*_ and *ρ_e_*_,*b*_ of both parts are known, the currents *i_a_* and *i_b_* in each part of the conductor can be calculated as,

*r_a_*(*t*) = *ρ_e_*_,*a*,*To*_ · [1 + *α*_a,AC_(*T*(*t*) − T_0_)]/*S_a_*, *r_b_*(*t*) = *ρ_e_*_,*b*,*To*_ · [1 + *α_b_*_,*AC*_(*T*(*t*) − T_0_)]/*S_b_* (Ω/m) (18)

*i_a_*(*t*) = *i*(*t*)·*r_b_*(*t*)/[*r_a_*(*t*) + *r_b_*(*t*)], *i_b_*(*t*) = *i*(*t*)·*r_a_*(*t*)/[*r_a_*(*t*) + *r_b_*(*t*)] (A) (19)

*J_a_*(*t*) = *i_a_*(*t*)/*S_a_*, *J_b_*(*t*) = *i_b_*(*i*)/*S_b_* (A/m^2^) (20)

## 4. Effective Radial Thermal Conductivity *k_r_*_,*eff*_, Its Determination and Published Values

### 4.1. Effective Radial Thermal Conductivity k_r,eff_

The heat generated in the inner layers of the conductor due to *i*^2^*r_AC_* is conducted radially along the contact surface from one strand to the next until it reaches the outer surface [22,34], so a radial thermal gradient is expected. When it reaches the conductor surface, this heat is dissipated to the surrounding atmospheric air primarily by convection and, to a lesser extent, by radiation. Under transient conditions, some of the heat may be stored in the conductor. The radial temperature difference between the center and the surface of the conductor depends on the conductor geometry (stranding and number of layers), the current level, the electrical resistance and the effective radial thermal conductivity [19].

**Figure 5:**
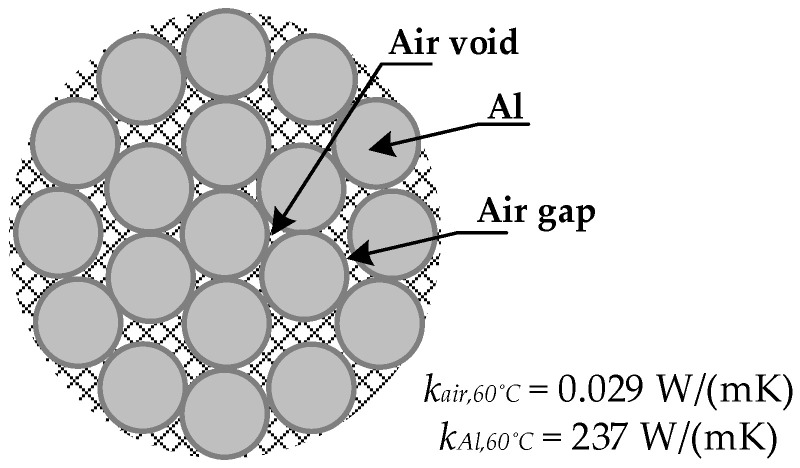
Cross-section of a stranded bare conductor.

As shown in Figure 5, transmission line conductors are not solid, but stranded, which makes the physics of the problem more complex.

According to [20,22], in multilayer stranded conductors, most of the *i*^2^*r_AC_* heat flow is conducted radially through the air voids that exist between strands in adjacent layers and through the air gaps that exist at the contact area between strands in adjacent layers, as shown in Figure 5. Internal convective and radiative effects are negligible, and the contact area between adjacent wires increases with the tension in the conductor [20]. The contact area depends on corrosion effects and accumulated grease. Therefore, the effective radial thermal conductivity *k_r_*_,*eff*_ (W/(mK)) of the conductor is greatly reduced and is much lower than the thermal conductivity of the aluminum material [35]. This is because the thermal conductivity of air is several orders of magnitude lower than that of the aluminum strands (see Figure 5), resulting in *k_r_*_,*eff*_ ~ *k_Al_*/100 due to the resistance to radial heat flow of the air voids and the interstrand contact [17]. It is also known that for constant current operation, *k_r_*_,*eff*_ increases (i.e., the radial temperature difference decreases) with increasing values of the mechanical axial tension, interlayer contact pressure and air pressure [22] and with a decreasing degree of corrosion [19]. Conductors with fewer layers and higher wind speeds also tend to increase *k_r_*_,*eff*_ [36].

Thermal conductivity measurements of stranded conductors in indoor and outdoor environments are extremely difficult due to the measurement variability associated with the measurement technique [36]. In real applications, the situation is even worse because there are many factors that affect *k_r_*_,*eff*_. In addition, it is impractical to measure the radial temperature distribution inside the conductor in real applications, so it is important to generate accurate conductor models that take into account the temperature distribution inside the conductor.

### 4.2. Determination of the Radial Thermal Conductivity k_r,eff_

According to several references [11,12,19], in the steady state, the radial temperature difference Δ*T*(*r*) is directly proportional to *P_internal_* = *i*^2^*r_AC_* and inversely proportional to the effective radial thermal conductivity *k_r_*_,*eff*_ as,

(21) $$\Delta T(r)=T(r)-T_{s}=\frac{i^{2}r_{AC}(T_{average})}{2\pi k_{r,eff}}\left[\frac{r_{s}^{2}-r^{2}}{2(r_{s}^{2}-r_{c}^{2})}+\frac{r_{c}^{2}}{r_{s}^{2}-r_{c}^{2}}\ln(r/r_{s})\right]$$

From (21), the effective thermal conductivity *k_eff_* of the conductor can be determined as [19],

(22) $$k_{r,eff}=\frac{i^{2}r_{AC}(T_{average})}{2\pi [T(r)-T_{s}]}\left[\frac{r_{s}^{2}-r^{2}}{2(r_{s}^{2}-r_{c}^{2})}+\frac{r_{c}^{2}}{r_{s}^{2}-r_{c}^{2}}\ln(r/r_{s})\right]$$

In the case of all aluminum conductors, since *r_c_* = 0, the above equations are simpler [17],

(23) $$\Delta T(r=0)=T(r=0)-T_{s}=\frac{i^{2}r_{AC}(T_{average})}{4\pi k_{r,eff}}$$

(24) $$k_{r,eff}=\frac{i^{2}r_{AC}(T_{average})}{4\pi [T(r=0)-T_{s}]}$$

From (24), *k_r_*_,*eff*_ can be determined if *i*^2^*r_AC_* and the total radial temperature gradient are known [20].

In [17], the following expression for the radial temperature gradient was obtained by solving the heat equation of a homogeneous and symmetric cylindrical conductor,

(25) $$\Delta T(r)=T(r)-T_{s}=\frac{i^{2}r_{AC}(T_{average})}{4\pi k_{r,eff}}\left[1-{\left(\frac{r}{r_{s}}\right)}^{2}\right]$$

which also leads to (23) when *r* = 0. Note that (23)–(25) assume that the electrical resistance of the conductor is constant throughout its cross-section despite the small radial thermal gradients.

### 4.3. Published Values of the Radial Thermal Conductivity k_r,eff_

As explained above, published works suggest that reasonable values of *k_r_*_,*eff*_ are in the range of 0.5–4.0 W/(mK), and in the case of ACSR conductors at moderate current densities, Refs. [11,12,35] suggest *k_r_*_,*eff*_ ≈ 2 W/(mK) for aluminum strands, while in the case of ACSR conductors under little or no applied tension, *k_r_*_,*eff*_ ≈ 1 W/(mK). Ref. [35] suggests that *k_r_*_,*eff*_ ≈ 1.5 W/(mK) for steel strands. A bibliographic review presented in [19] analyzing various conductors suggests values of *k_r_*_,*eff*_ in the range of 1.2–10.0 W/(mK). Analyzing an all-aluminum conductor (AAC) with an outer diameter of 44.45 mm and 91 aluminum strands of 4.04 mm each, Morgan [22] suggested that *k_r_*_,*eff*_ ≈ 0.7–1.1 W/(mK) under axial tensions of 4.45–22.2 kN. In [37], values of *k_r_*_,*eff*_ = 0.98 W/(mK) are reported for a Cardinal ACSR conductor and *k_r_*_,*eff*_ = 4 W/(mK) for a Marigold AAC conductor. In [19], values of *k_r_*_,*eff*_ ≈ 1.34–1.90 W/(mK) are reported for different types of copper conductors, *k_r_*_,*eff*_ ≈ 2.10–2.86 W/(mK) for different AAC conductors, *k_r_*_,*eff*_ ≈ 1.86–4.47 W/(mK) for different AAAC conductors, and *k_r_*_,*eff*_ ≈ 2.46–4.70 W/(mK) for different ACSR conductors.

The value of *k_r_*_,*eff*_ is strongly influenced by the factors that determine the amount of air trapped between the strands, such as conductor topology, number of layers, strand geometry (round versus trapezoidal) or conductor temperature, since an excessive temperature will cause separation between adjacent strands, known as birdcaging [35]. For a specific application, its value can be approximated from the tabulated values or by fitting experimental test data to simulation results obtained with the radial model proposed in this paper.

For stranded conductors operating below the SLR limit, the temperature gradient between the surface and the center of the conductor is much smaller than the conductor temperature increase over the ambient temperature. However, below the DLR limit, and for new applications such as HTLS conductors, the radial temperature gradients may not be negligible [17].

## 5. Radial Problem Formulation

This section develops the equations to solve the problem using a one-dimensional radial formulation.

### 5.1. Analysis of Node i = 1 Belonging to the Core (Material a)

If the core is conductive, node *i* = 1 generates heat due to the internal losses, which is conducted outward to node *i* = 2. The discrete form of the transient heat transfer Equation (16) applied to node *i* = 1 is as follows,

(26) $$\rho_{a}\pi {\left(x_{1}+0.5\Delta x_{a}\right)}^{2}c_{p,a}\Delta x_{a}\frac{T_{1}^{j+1}-T_{1}^{j}}{\Delta t\Delta x_{a}}=J_{i,a}^{2}S_{i,a}\rho_{e,a}(T_{i}^{j})-\frac{k_{a}2\pi (x_{1}+0.5\Delta x_{a})(T_{1}^{j+1}-T_{2}^{j+1})}{\Delta x_{a}}$$

The terms in (26) can be rearranged as needed in the TDMA formulation (11), so that for the first node becomes

(27) $$a_{1}=0,\;b_{1}=\frac{\rho_{a}\pi {\left(x_{1}+0.5\Delta x_{a}\right)}^{2}c_{p,a}}{\Delta t}+\frac{k_{a}2\pi (x_{1}+0.5\Delta x_{a})}{\Delta x_{a}},\;c_{1}=-\frac{k_{a}2\pi (x_{1}+0.5\Delta x_{a})}{\Delta x_{a}},\;d_{1}=\frac{\rho_{a}\pi {\left(x_{1}+0.5\Delta x_{a}\right)}^{2}c_{p,a}T_{1}^{j}}{\Delta t}+J_{i,a}^{2}S_{i,a}\rho_{e,a}(T_{i}^{j})$$

For conductors with a non-conducting core, such as aluminum conductor composite core (ACCC) conductors, *J_i_*_,*a*_ = 0 must be used in (26) and (27).

### 5.2. Analysis of a Generic Node 2 ≤ i ≤ N_a_ Belonging to the Core (Material a)

This generic node has an area *S_i_*_,*a*_ = π(*x_i_* + 0.5Δ*x_a_*)^2^ − π(*x_i_* − 0.5Δ*x_a_*)^2^ = 2π*x_i_*Δ*x_a_*. When evaluating the heat transfer in this generic node *i*, we must take into account the heat generation term, the heat input heat conducted from the left or inner element and the output heat conducted to the right or outer element, resulting in,

(28) $$\rho_{a}2\pi x_{i}\Delta x_{a}c_{p,a}\Delta x_{a}\frac{T_{i}^{j+1}-T_{i}^{j}}{\Delta t\Delta x_{a}}=J_{i,a}^{2}S_{i,a}\rho_{e,a}(T_{i}^{j})+\frac{k_{a}2\pi (x_{i}-0.5\Delta x_{a})(T_{i-1}^{j+1}-T_{i}^{j+1})}{\Delta x_{a}}-\frac{k_{a}2\pi (x_{i}+0.5\Delta x_{a})(T_{i}^{j+1}-T_{i+1}^{j+1})}{\Delta x_{a}}$$

The TDMA coefficients are obtained from (28) as,

(29) $$a_{i}=-\frac{k_{a}2\pi (x_{i}-0.5\Delta x_{a})}{\Delta x_{a}},\;b_{i}=\frac{\rho_{a}2\pi x_{i}\Delta x_{a}c_{p,a}}{\Delta t}+\frac{k_{a}4\pi x_{i}}{\Delta x_{a}},\;c_{i}=-\frac{k_{a}2\pi (x_{i}+0.5\Delta x_{a})}{\Delta x_{a}},\;d_{i}=\frac{\rho_{a}2\pi x_{i}\Delta x_{a}c_{p,a}T_{i}^{j}}{\Delta t}+J_{i,a}^{2}S_{i,a}\rho_{e,a}(T_{i}^{j})$$

### 5.3. Analysis of the Boundary Node between the Core and the Conductor (i = N_a_)

The formulation of this node is similar to that in (28), taking into account the different thermal conductivities (*k_a_* and *k_b_*) and electrical resistivities (*ρ_e_*_,*a*_ and *ρ_e_*_,*b*_) on either side of the boundary,

(30) $$\rho_{a}S_{i,a}c_{p,a}\frac{T_{i}^{j+1}-T_{i}^{j}}{\Delta t}+\rho_{b}S_{i,b}c_{p,b}\frac{T_{i}^{j+1}-T_{i}^{j}}{\Delta t}=J_{i,a}^{2}S_{i,a}\rho_{e,a}(T_{i}^{j})+J_{i,b}^{2}S_{i,b}\rho_{e,b}(T_{i}^{j})+\frac{k_{a}2\pi (x_{i}-0.5\Delta x_{a})(T_{i-1}^{j+1}-T_{i}^{j+1})}{\Delta x_{a}}-\frac{k_{b}2\pi (x_{i}+0.5\Delta x_{b})(T_{i}^{j+1}-T_{i+1}^{j+1})}{\Delta x_{b}}$$

where *S_i_*_,*a*_ = π(*x_i_*)^2^ − π(*x_i_* − 0.5Δ*x_a_*)^2^ and *S_i_*_,*b*_ = π(*x_i_* + 0.5Δ*x_b_*)^2^ − π(*x_i_*)^2^. The TDMA coefficients in this boundary are as follows,

(31) $$\begin{array}{l} a_{i}=-\frac{k_{a}2\pi (x_{i}-0.5\Delta x_{a})}{\Delta x_{a}},\;b_{i}=\frac{\rho_{a}S_{i,a}c_{p,a}}{\Delta t}+\frac{\rho_{b}S_{i,b}c_{p,b}}{\Delta t}+\frac{k_{a}2\pi (x_{i}-0.5\Delta x_{a})}{\Delta x_{a}}+\frac{k_{b}2\pi (x_{i}+0.5\Delta x_{b})}{\Delta x_{b}},\\ c_{i}=-\frac{k_{b}2\pi (x_{i}+0.5\Delta x_{b})}{\Delta x_{b}},\;d_{i}=\frac{\rho_{a}S_{i,a}c_{p,a}T_{i}^{j}}{\Delta t}+\frac{\rho_{b}S_{i,b}c_{p,b}T_{i}^{j}}{\Delta t}+J_{i,a}^{2}S_{i,a}\rho_{e,a}(T_{i}^{j})+J_{i,b}^{2}S_{i,b}\rho_{e,b}(T_{i}^{j}) \end{array}$$

In the case of single-material conductors, such as all-aluminum alloy conductors (AAAC), *k_a_* = *k_b_*, *c_p_*_,*a*_ = *c_p_*_,*b*_ and *ρ_e_*_,*a*_ = *ρ_e_*_,*b*_.

### 5.4. Analysis of a Generic Node N_a_ + 1 ≤ i ≤ N_a_ + N_b_ Belonging to the Conductor (Material b)

This formulation is similar to that in the generic node belonging to the core (2 ≤ *i* ≤ *N_a_*), but the subscripts *a* are replaced by subscripts *b*,

(32) $$\rho_{b}2\pi x_{i}\Delta x_{b}c_{p,b}\Delta x_{b}\frac{T_{i}^{j+1}-T_{i}^{j}}{\Delta t\Delta x_{b}}=J_{i,b}^{2}S_{i,b}\rho_{e,b}(T_{i}^{j})+\frac{k_{b}2\pi (x_{i}-0.5\Delta x_{b})(T_{i-1}^{j+1}-T_{i}^{j+1})}{\Delta x_{b}}-\frac{k_{b}2\pi (x_{i}+0.5\Delta x_{b})(T_{i}^{j+1}-T_{i+1}^{j+1})}{\Delta x_{b}}$$

The TDMA coefficients are obtained from (32) as,

(33) $$a_{i}=-\frac{k_{b}2\pi (x_{i}-0.5\Delta x_{b})}{\Delta x_{b}},\;b_{i}=\frac{\rho_{b}2\pi x_{i}\Delta x_{b}c_{p,b}}{\Delta t}+\frac{k_{b}4\pi x_{i}}{\Delta x_{b}},\;c_{i}=-\frac{k_{b}2\pi (x_{i}+0.5\Delta x_{b})}{\Delta x_{b}},\;d_{i}=\frac{\rho_{b}2\pi x_{i}\Delta x_{b}c_{p,b}T_{i}^{j}}{\Delta t}+J_{i,b}^{2}S_{i,b}\rho_{e,b}(T_{i}^{j})$$

### 5.5. Analysis of the Boundary Node between the Conductor and the Air (i = N_a_ + N_b_ + 1)

Assuming Δ*x_air_* = Δ*x_b_*, this boundary node has an area *S_i_*_,*a*_ = π(*x_i_*)^2^ − π(*x_i_* − 0.5Δ*x_b_*)^2^ = πΔ*x_air_*(*x_i_* − 0.25Δ*x_b_*), as shown in Figure 3. This is the outermost node, which is placed in the boundary between the conductor and the air, so in the absence of wind (worst case), heating by solar radiation and radiative and convective cooling effects must be considered, resulting in,

(34) $$\begin{array}{c} \rho_{b}\pi \Delta x_{b}(x_{i}-0.25\Delta x_{b})c_{p,b}\Delta x_{b}\frac{T_{i}^{j+1}-T_{i}^{j}}{\Delta t\Delta x_{b}}=\\ =\frac{k_{b}2\pi (x_{i}-0.5\Delta x_{b})(T_{i-1}^{j+1}-T_{i}^{j+1})}{\Delta x_{b}}+J_{i,b}^{2}S_{i,b}\rho_{e,b}(T_{i}^{j})+\alpha Q_{sr}D_{c}-h\pi D_{c}(T_{i}^{j+1}-T_{air})-\varepsilon \sigma \pi D_{c}[{\left(T_{i}^{j}\right)}^{4}-{\left(T_{air}\right)}^{4}] \end{array}$$

Finally, the TDMA coefficients of the outermost boundary node are as follows,

(35) $$\begin{array}{c} a_{i}=-\frac{k_{b}2\pi (x_{i}-0.5\Delta x_{b})}{\Delta x_{b}},\;b_{i}=\frac{\rho_{b}\pi \Delta x_{b}(x_{i}-0.25\Delta x_{b})c_{p,b}}{\Delta t}+\frac{k_{b}2\pi (x_{i}-0.5\Delta x_{b})}{\Delta x_{b}}+h\pi D_{c}\\ c_{i}=0,\;d_{i}=\frac{\rho_{b}\pi \Delta x_{b}(x_{i}-0.25\Delta x_{b})c_{p,b}T_{i}^{j}}{\Delta t}+J_{i,b}^{2}S_{i,b}\rho_{e,b}(T_{i}^{j})+\alpha Q_{sr}D_{c}+h\pi D_{c}T_{air}-\varepsilon \sigma \pi D_{c}[{\left(T_{i}^{j}\right)}^{4}-{\left(T_{air}\right)}^{4}] \end{array}$$

This code was programmed by the authors of this paper using Matlab^®^ R2022b.

## 6. Conductors Analyzed and Experimental Setup

To validate and evaluate the accuracy of the proposed radial model, this paper analyzes three different conductors, an all-aluminum alloy conductor (AAAC), an ACSR conductor and a high-temperature low-sag (HTLS) conductor, which are described in the following sections.

### 6.1. AAAC Conductors

AAAC conductors use a high-strength aluminum alloy to achieve good sag characteristics and a high strength-to-weight ratio. All strands are made of high-strength aluminum alloy, so there is no steel core. AAAC conductors offer higher corrosion resistance compared to ACSR conductors.

**Figure 6:**
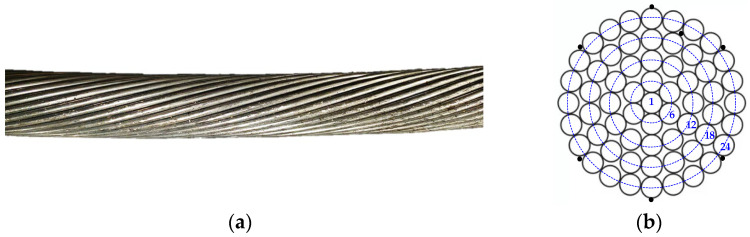
Aster 570 AAAC conductor with the position of the thermocouples (black dots). (**a**) Photograph of the conductor. (**b**) Distribution of the aluminum alloy strands (1/6/12/18/24).

This paper analyzes the thermal behavior of the Aster 570 AAAC conductor, which has 61 aluminum strands and a 1/6/12/18/24 distribution, as shown in Figure 6.

Table 1 shows the main parameters of the Aster 570 AAAC conductor.

### 6.2. ACSR Conductors

As explained above, ACSR conductors consist of multiple layers of aluminum strands wound helically around an inner core made of galvanized steel strands. While the aluminum strands carry most of the electrical current, the steel strands provide the mechanical strength. This paper also analyzes the thermal behavior of a two-layer ACSR conductor (Partridge 135-AL1/22-ST1A, EMTA Kablo, Istanbul, Turkey). This conductor has a steel core with seven steel strands (1/6 steel configuration) and two conductive aluminum layers with 10 and 16 aluminum strands (10/16 aluminum configuration).

**Figure 7:**
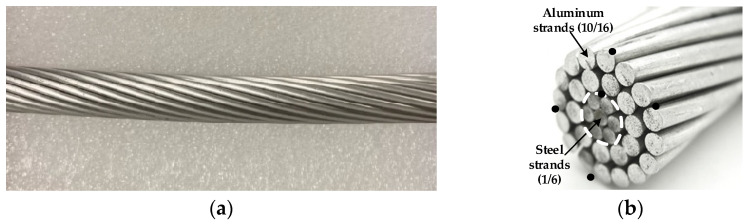
Partridge 135-AL1/22-ST1A ACSR conductor with the location of the thermocouples (black dots). (**a**) Photograph of the conductor. (**b**) Strand distribution (1/6 steel, 10/16 aluminum).

Figure 7 shows the Partridge 135-AL1/22-ST1A ACSR conductor.

Table 2 shows the main parameters of the Partridge 135-AL1/22-ST1A ACSR conductor, which consists of 7 steel strands with a 1/6 distribution and 26 aluminum strands with a 10/16 distribution.

### 6.3. HTLS Conductors

**Figure 8:**
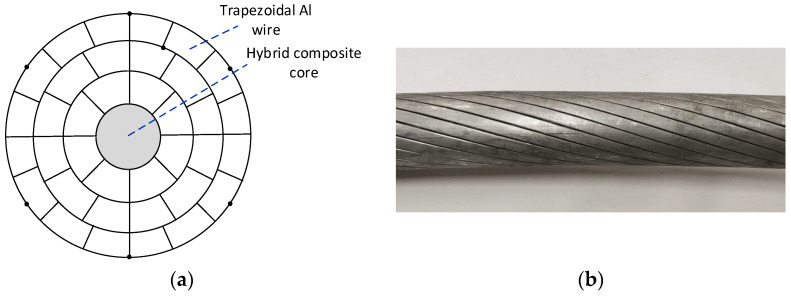
(**a**) Cross-section of the Dhaka ACCC/TW conductor with a non-conducting hybrid composite core. (**b**) Photograph of the conductor.

An HTLS conductor-type aluminum conductor composite core (ACCC) with trapezoidal wire (TW), also known as ACCC/TW, was analyzed. To limit sag at high temperatures, this HTLS conductor has a non-conductive, lightweight and high-strength hybrid core of carbon and glass fibers embedded in a resin matrix [38]. Fully annealed and helically wound trapezoidal aluminum strands surround the hybrid core. These fully annealed aluminum strands are purer and more conductive than the strands used in conventional ACSR conductors. The continuous operating temperature of these conductors is approximately 180 °C [39]. Figure 8 shows the cross-section of the ACCC/TW used in this work and the location of the thermocouples (black dots).

Table 3 shows the main parameters of the three-layer Dhaka ACCC/TW conductor.

### 6.4. Experimental Setup

The tests were performed in an indoor high-current laboratory. All tested conductors were connected to the output of the high-current transformer (0–10 V output voltage, 0–10 kA output current), forming a low-impedance loop.

Since the experiments were conducted indoors, LED projectors (Series AGRO-K, average photosynthetic photon flux density 430 μMol/m^2^/s, Venalsol, Valencia, Spain) were used to simulate the solar irradiance. The global solar irradiance was measured with a global radiation meter (PCE-SPM 1, silicon sensor, 400–1100 nm, 0–2000 W/m^2^, 0.1 W/m^2^, accuracy ± 10 W/m^2^, PCE Instruments, Southampton, UK).

The AC resistance per unit length *r*_AC_ of the conductors was determined experimentally from (5) by measuring the voltage drop across a 1 m length of conductor and the phase shift between the voltage drop and the current.

The current flowing through the loop was measured with a Rogowski coil (CWT500LFxB, 0.06 mV/A sensitivity, ±1% current accuracy, PEM, Nottingham, UK). The data provided by the Rogowski coil and the voltage drop in the conductors were acquired using an NI USB-6343 data acquisition card (National Instruments, Dallas, TX, USA), from which the phase shift between the voltage drop and the current can be determined, and thus the AC resistance per unit length *r*_AC_.

Low inertia T-type thermocouples were used to measure the surface temperature of the conductors. The data provided by the thermocouples was acquired using a thermocouple input module (TC08, ±1 °C, Omega, Northbank, Manchester, UK).

A Python code was used to synchronize the measurements taken with the TC08 thermocouple input module and the NI USB-6343 data acquisition card.

**Figure 9:**
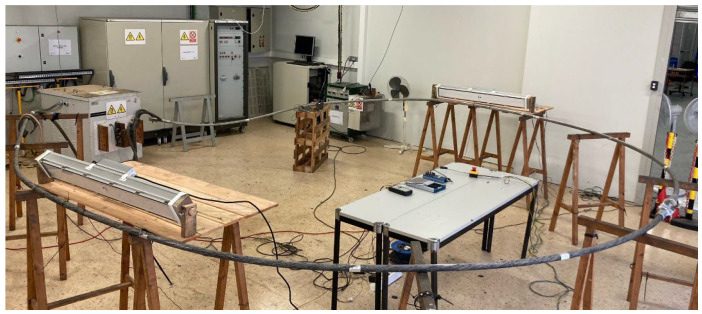
Experimental setup, including the high-current transformer, the conductor loop, the LED lamps and the thermocouples.

The experimental setup is shown in Figure 9.

To avoid heat sink effects from the edges, the conductors were long enough (about 8–10 m each) and measurements were made in the center of the conductor [17].

Different thermocouples were placed on the outer surface of each conductor to determine the average surface temperature. To measure the internal temperature, thermocouples were also implanted directly into different layers of the conductor, forcing adjacent strands to open and taking care not to deform the geometry of the conductor once in place. Thermal paste was used to improve the thermal contact between the thermocouples and the aluminum strands. The test setup was located indoors, which allowed measurements to be made at zero wind speed.

## 7. Radial Temperature Profile and Model Validation from Experimental Results

This section presents experimental results, which are used to validate the simulation model presented in Section 5.

### 7.1. ASTER 570 AAAC Conductor

**Figure 10:**
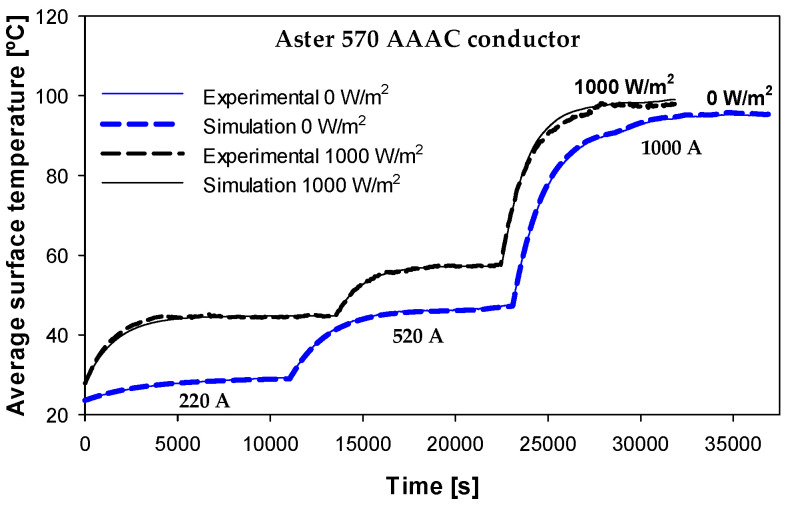
Surface temperature. Experimental and simulation results obtained with the Aster 570 AAAC conductor at a solar irradiance of 1000 W/m^2^. The average difference between the experimental and simulation results was 0.47% at 0 W/m^2^ and 0.84% at 1000 W/m^2^.

This section validates the simulation model with experimental results using the ASTER 570 AAAC conductor. Simulation models are attractive because they can provide results very close to experimental results in a faster, less expensive and more environmentally friendly manner. Figure 10 compares the experimental and simulation results obtained with the Aster 570 conductor using different currents (220–520–1000 A) and a solar irradiance of 0 W/m^2^ and 1000 W/m^2^.

The results presented in Figure 10 show a great similarity between the experimental and simulation results. Although two solar irradiance conditions were tested and simulated, i.e., 0 W/m^2^ and 1000 W/m^2^, in both cases, the simulation results were very close to the experimental ones, which allows us to validate the simulation model presented in Section 5. It should be noted that Figure 10 shows the average readings of the different thermocouples placed on the surface of the conductor. It should be noted that the proposed radial model assumes a uniform surface temperature distribution, although the laboratory results indicate that the higher temperature corresponds to the lowest point, while the lower temperature of the surface corresponds to the highest point.

**Figure 11:**
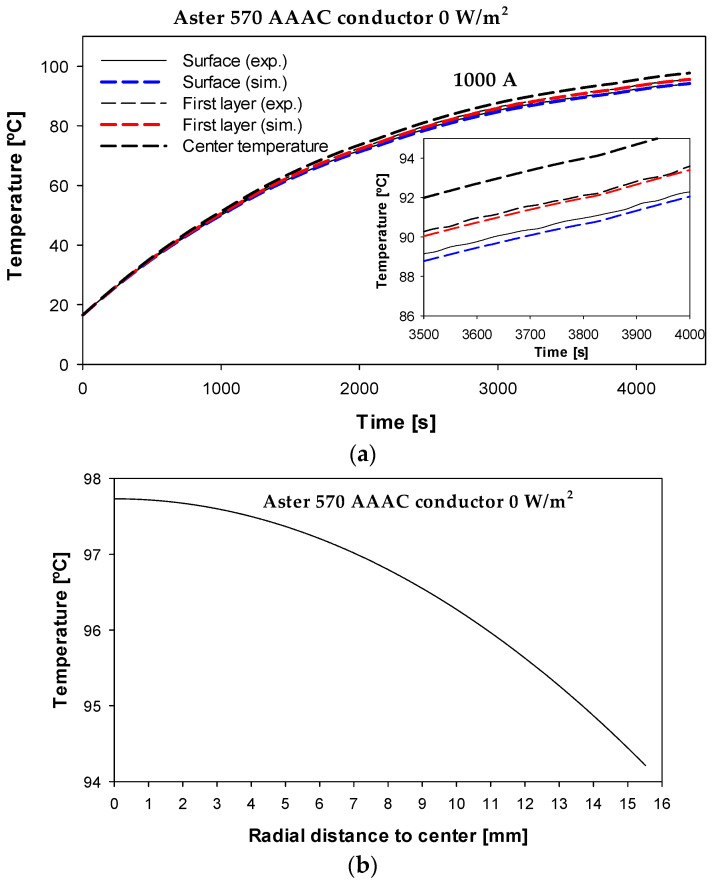
Aster A570 with different thermocouples on the surface and one thermocouple in the interface between the two outermost layers. (**a**) Experimental and simulated temperatures. (**b**) Predicted radial temperature profile.

To further validate the simulation model, the surface temperature and the temperature at the interface between the outer layer and the second layer were measured when a step current of 1000 A was applied without solar radiation. The experimental and simulated results are shown in Figure 11. Note that the experimental value of the surface temperature is the average of the readings from the different thermocouples placed on the outer surface.

The results shown in Figure 11a again show a strong similarity between experimental and simulation results. The results shown in Figure 11b predict a temperature gradient of about 3.5 °C between the center and the surface of the conductor under the operating conditions shown in Figure 11, while the calculated temperature difference from (25) was 3.4 °C. The average temperature difference between the experimental results and the simulation results was 0.32% and 0.38% for the surface and first layer temperatures, respectively.

### 7.2. Partridge 135-AL1/22-ST1A ACSR Conductor

**Figure 12:**
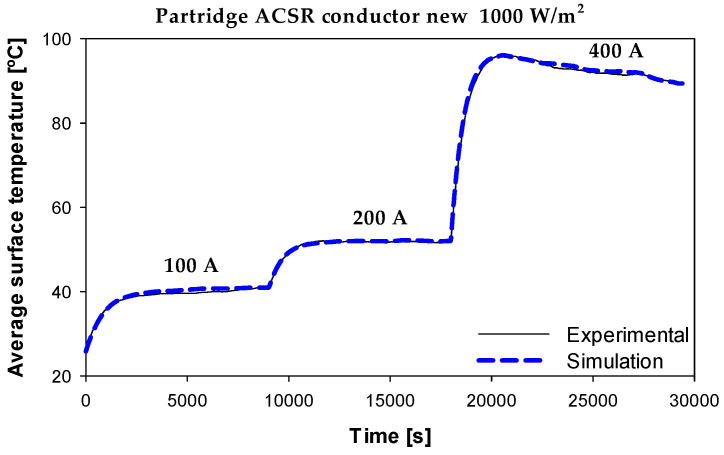
Surface temperature. Experimental and simulation results with the Partridge 135-AL1/22-ST1A ACSR conductor at 1000 W/m^2^ of solar irradiance. The average temperature difference between the experimental and simulation results was 0.75%.

Further validation of the simulation model was performed on the 135-AL1/22-ST1A ACSR conductor. Figure 12 compares the experimental and simulation results obtained with the Aster 570 conductor at different currents (100–200–400 A) and 1000 W/m^2^ of solar irradiance.

The results shown in Figure 12 also show a great similarity between the experimental and simulation results. These results are particularly relevant due to the inherent complexity of ACSR conductors composed of two materials, i.e., the magnetic core of galvanized steel strands and the aluminum strands. These results further validate the accuracy of the proposed radial model.

**Figure 13:**
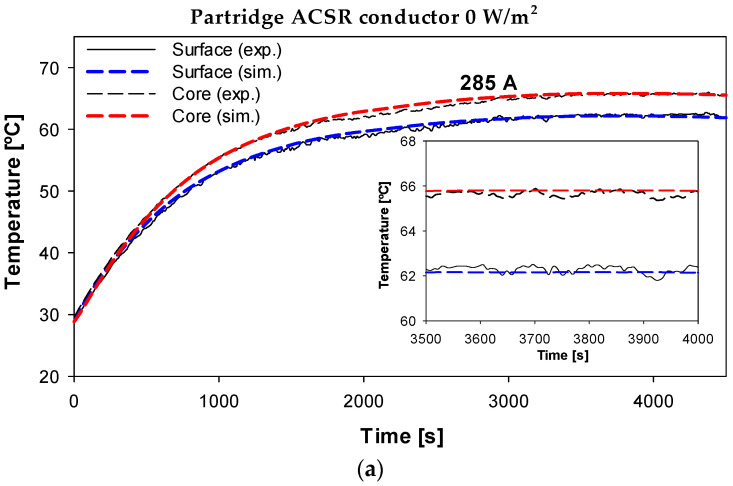

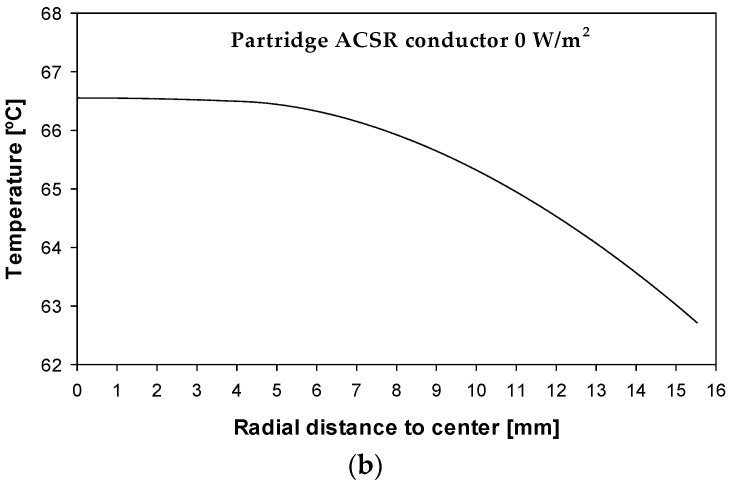
Partridge ACSR conductor (*ε* = 0.26) with thermocouples in the interface between the second layer and the surface. (**a**) Experimental and simulated temperatures. (**b**) Predicted radial temperature profile.

Next, the surface temperature and the temperature at the interface between the second layer and the core were measured when a step current of 285 A was applied in the absence of solar radiation. The experimental and simulated results are shown in Figure 13, which again shows a great similarity between the experimental and simulated results.

The results shown in Figure 13b predict a temperature gradient of about 3.8 °C between the core and the surface of the conductor under the operating conditions shown in Figure 13, while the calculated temperature difference from (21) was 3.6 °C. The average temperature difference between the experimental and simulation results was 0.61% and 0.54% for the surface and core temperatures, respectively.

### 7.3. Dhaka HTLS Conductor

**Figure 14:**
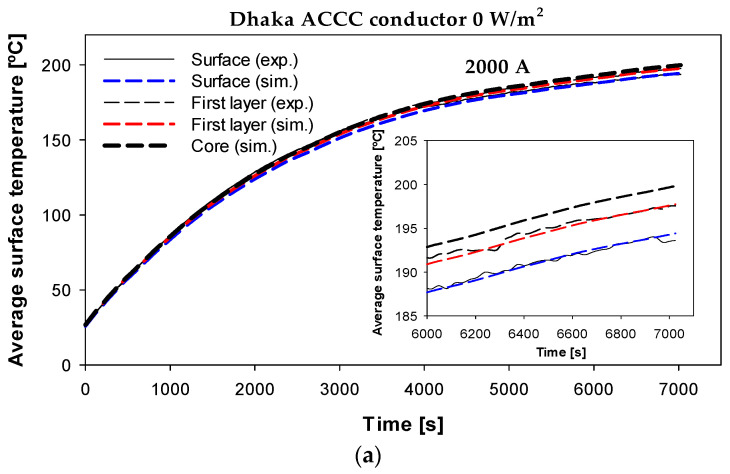

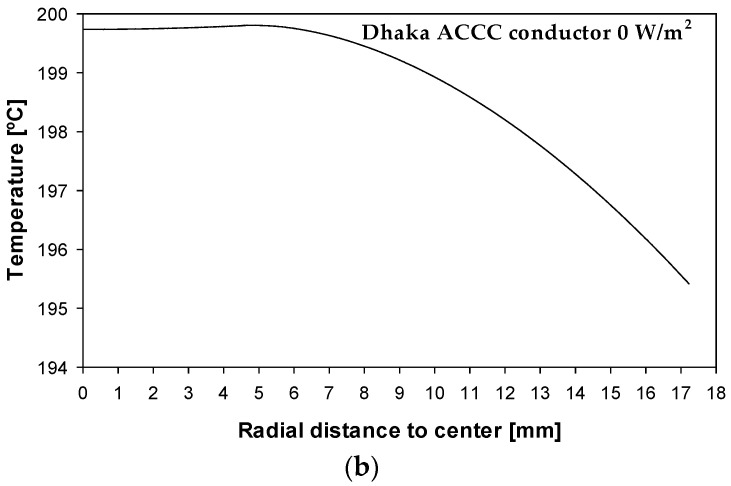
Dhaka HTLS conductor (*ε* = 0.40) with thermocouples in the interface between the first and second outermost layers and the surface. (**a**) Experimental and simulated temperatures. (**b**) Predicted radial temperature profile.

Finally, to further validate the simulation model for high-temperature operations, the Dhaka HTLS conductor was tested under a step current of 2000 A. The surface temperature and the temperature at the interface between the first and second outermost layers were measured. The experimental and simulated results are presented in Figure 14, which again shows a great similarity between the experimental and simulated results, even at high-temperature operation.

The results shown in Figure 14b predict a temperature gradient of about 4.4 °C between the core and the surface of the conductor under the operating conditions shown in Figure 14, almost the same temperature difference calculated from (21). The average temperature difference between experimental and simulation results was 1.13% and 1.00% for the surface and first layer temperatures, respectively. It should be noted that ACCC conductors are challenging because they operate at very high temperatures and have a non-conductive hybrid composite core. Nevertheless, the proposed radial model reproduces the experimental laboratory results with high accuracy.

### 7.4. Comparative Results of the Proposed Radial Model versus the Classical Model

This section analyzes the accuracy of the proposed radial method in comparison with the classical model that assumes a homogeneous temperature inside the conductor, which is equivalent to assuming infinite thermal conductivity of the conductor material. The results obtained are summarized in Table 4.

The results presented in Table 4 show that although both models are accurate, the radial model gives results more similar to the experimental ones for the three conductors analyzed in this work operating under different conditions.

Finally, it should be emphasized that in order to show the accuracy of the proposed model, three types of conductors with very different characteristics have been analyzed, namely an all-aluminum alloy conductor (AAAC), an ACSR conductor and an ACCC (HTLS) conductor. The AAAC conductor is composed of aluminum strands only, while the two other two conductors are composed of two materials. While ACSR conductors have a magnetic core made of galvanized steel strands, ACCC conductors have a non-conductive hybrid composite core. Despite the different types of conductors, the different solar radiation conditions, the different current densities studied and the different temperature ranges achieved, the simulation results obtained with the proposed radial model are very close to the experimental laboratory results. Therefore, it can be concluded that this radial model can be effectively used for the purposes of DLR.

## 8. Conclusions

Stranded conductors are critical elements of overhead power lines. A radial thermal gradient is created as the internal heat generated is conducted to the outer surface. In order to fully utilize the current-carrying capacity of the conductor and to ensure safe operation, e.g., when developing accurate conductor line models to apply dynamic line rating (DLR) strategies, this effect must be taken into account because two important terms of the heat transfer equation—i.e., the internal heating, which is the main source of heating, and the stored heat, which strongly affects the dynamics of the heat transfer—depend on the internal temperature distribution. In addition, annealing, creep and sag effects are strongly influenced by the radial temperature distribution within the conductor. Therefore, accurate thermoelectric conductor models must take into account the temperature distribution within the conductor.

This paper has presented a radial one-dimensional thermoelectric model for bare-stranded conductors. The accuracy of the proposed model has been evaluated through experimental tests performed on three types of conductors, namely AAAC, ACSR and ACCC-HTLS conductors. The experimental results presented in this paper show that the thermal gradient between the center and the outer surface of the conductor can be predicted with accuracy using the proposed radial one-dimensional model, which improves the capabilities of the models proposed in the IEC, Cigré and IEEE standards, especially when designing DLR approaches. In particular, the results presented under different conditions (varying current density, solar irradiance and temperature range) show that the average differences between the laboratory results and the results of the radial model are usually less than 1%, which proves the accuracy of the proposed model. The results presented in this paper have also shown the superior accuracy of the proposed radial model with respect to the results of the model that considers a homogeneous temperature distribution inside the conductor.

## Figures and Tables

**Table 1:** Main parameters of the Aster 570 AAAC conductor.

Input Parameters	Units	Aster 570 AAAC Conductor
Number of strands	(-)	61
Strand diameter	(mm)	3.45
Outer diameter	(mm)	31.05
Effective conductor cross-section	(mm^2^)	570.22
Conductor emissivity	(-)	0.25
Effective radial thermal conductivity	(W/(mK))	2.0
AC resistance per unit length at 20 °C	(Ω/km)	0.06763
Temperature coefficient of resistance	(K^−1^)	0.0036
Density of conductor material	(kg/m^3^)	2700 (Al)
Mass per unit length	(kg/m)	1.576
Emissivity	(-)	0.25

**Table 2:** Main parameters of the new two-layer Partridge 135-AL1/22-ST1A ACSR conductor.

Description	Unit	Value
AC resistance per unit length at 20 °C	(Ω/km)	0.2038
Temperature coefficient of resistance	(K^−1^)	0.0035
Current carrying capacity	(A)	430
Number of aluminum strands	(-)	26 (10/16)
Number of steel strands	(-)	7 (1/6)
Aluminum strand diameter	(mm)	2.57
Steel wire diameter	(mm)	2.0
Outer diameter	(mm)	16.3
Aluminum area	(mm^2^)	134.9
Steel area	(mm^2^)	22.0
Mass per unit length	(kg/m)	0.5445
Core mass per unit length	(kg/m)	0.1728
Aluminum mass per unit length	(kg/m)	0.3717
Effective radial thermal conductivity of Al part	(W/(mK))	2.0
Effective radial thermal conductivity of steel part	(W/(mK))	1.5
Emissivity	(-)	0.26

**Table 3:** Main parameters of the three-layer Dhaka ACCC/TW conductor.

Description	Unit	Two-Layer
AC resistance per unit length at 20 °C	(Ω/km)	0.03984
Temperature coefficient of resistance	(K^−1^)	0.0040
Current carrying capacity	(A)	2000
Number of aluminum strands	(-)	36 (8/12/16)
Outer diameter	(mm)	34.45
Core diameter	(mm)	9.53
Aluminum area	(mm^2^)	723.9
Mass per unit length	(kg/m)	2.137
Core mass per unit length	(kg/m)	0.132
Aluminum mass per unit length	(kg/m)	2.005
Effective radial thermal conductivity	(W/(mK))	4.0
Emissivity	(-)	0.4

**Table 4:** Average difference between the laboratory experimental results and simulation results obtained with the proposed radial model and the classical homogeneous model.

Conductor	Data Origin	Average Difference	
		Radial Model	Classical Model
Aster A570 AAAC	Figure 11	0.32%	0.40%
Partridge ACSR	Figure 13	0.61%	0.97%
Dhaka ACCC/TW	Figure 14	1.13%	1.20%

## Data Availability

The data presented in this study are available on request from the corresponding author.
